# A Case of Myxedema Edema in a Patient With Factors XI and XII Deficiency and Sepsis: Unveiling Complications and Management Strategies

**DOI:** 10.7759/cureus.62961

**Published:** 2024-06-23

**Authors:** Leidhy Montecinos, Uma Gupta, Jacob A Kashani, Shachi Paudel, Khiet T Nguyen

**Affiliations:** 1 Medicine, Interfaith Medical Center, Brooklyn, USA; 2 Internal Medicine, One Brooklyn Health/Interfaith Medical Center, Brooklyn, USA; 3 Internal Medicine, Interfaith Medical Center, Brooklyn, USA

**Keywords:** thyroxine, myxedema coma, hypothyroidism, hypothermia, general edema

## Abstract

Hypothermic patients are rarely encountered in the emergency department (ED), indicating a potentially critical condition requiring immediate attention and diagnosis. Myxedema coma, a severe complication of hypothyroidism, presents as profound hypothermia and demands early recognition and proper treatment. We report the case of a 77-year-old female with no prior medical history of hypothyroidism. She presented to the ED with a one-and-a-half-month history of weakness, hypothermia, decreased mental status, and edema. Laboratory analysis confirmed hypothyroidism, leading to a diagnosis of myxedema coma. Treatment with thyroxine and glucocorticoid supplements resulted in a favorable outcome without complications. In conclusion, myxedema coma should be considered in hypothermic patients with altered mental status, even without a history of hypothyroidism. Prolonged hypothyroidism or acute events like sepsis, cerebrovascular accidents, gastrointestinal bleeding, cold exposure, trauma, or certain medications can precipitate this condition, emphasizing the need for prompt treatment initiation.

## Introduction

Myxedema coma is infrequent, with a prevalence of 0.22 cases per million yearly, but it poses a considerable mortality risk (25-60%) with factors such as advanced age, cardiac complications, impaired consciousness, persistent hypothermia, sepsis, and delayed treatment contributing to poor prognosis if not promptly identified and managed [1-4]. Myxedema coma is undoubtedly an endocrine emergency requiring prompt recognition and intervention. A frequently overlooked contributing factor to the myxedema crisis involves the cessation of thyroid supplements in critically ill patients. Myxedema coma can be triggered by sepsis, strokes, heart attacks, bleeding, cold exposure, trauma, or specific medications like lithium, amiodarone, and opioids. Additionally, hypothyroidism patients may develop congestive heart failure even without prior heart issues. Diagnosis relies on clinical symptoms and history, necessitating immediate treatment regardless of thyroid function test results [1]. Dutta et al. investigated 23 patients experiencing myxedema crisis and discovered that 39% of them had undiagnosed hypothyroidism detected only during the crisis. Sepsis emerged as the predominant precipitating factor, with a significant portion of patients (61%) having discontinued their thyroid supplements [5]. Reinhardt and Mann documented hypoxemia in 80%, hypercapnia in 54%, and hypothermia with temperatures below 94°F in 88% of all myxedema crisis patients [6].

This report presents a case involving hypothermia with myxedema coma and sepsis, alongside factor deficiency, encountered in the emergency department (ED). The patient's treatment was complicated by her inability to take oral medications, necessitating an extended period of intensive care.

## Case presentation

A 77-year-old female was brought to the ED due to hypothermia, leg swelling, and generalized weakness lasting for one month. On admission, the patient was drowsy and bradycardic with a heart rate of 48 and temperature of 32.3° centigrade at triage, and she had a Glasgow Coma Scale score of 10 out of 15 at presentation. Furthermore, she had generalized edema, an increased BMI of 30.6, and diffuse hair loss were also noted. She did not have a fever, chills, cough, chest pain, cold sweating, poor appetite, hemiparesis, bleeding, trauma, substance use, or exposure to cold. She has a history of pancreatic cancer status post-surgery on remission, and she had leg cellulitis with a blood culture positive for *Klebsiella pneumoniae* and a wound culture from a left leg ulcer positive for methicillin-resistant *Staphylococcus aureus*. 

In addition to hypothermia, her vital signs showed respiratory rate of 18 bpm, pulse of 48 bpm, and blood pressure of 118/53 mmHg. Her physical assessment indicated pale conjunctiva, widespread edema, and decreased reflexes. She was negative for thyromegaly and had a normal brownish stool upon digital rectal examination. The initial blood tests showed blood sugar of 129 mg/dL, white blood count (WBC) of 14000/uL (normal: 3500-11000/uL), hemoglobin (Hb) of 9.2 g/dL (normal: 12-16 g/dL), C-reactive protein of 80 mg/L (normal: <5 mg/L), aspartate transaminase (AST) of 64 U/L (normal: ≤34 U/L), sodium of 139 mEq/L (normal: 134-148 mEq/L), potassium of 2.6 mEq/L (normal: 3.6-5.0 mEq/L), and Cr of 1.5. Additional blood tests showed troponin-I, albumin of 2 g/dL, calcium of 7.8 mg/dL, cortisol of 12.1 ug/dL, and CEA of 26.6 ng/mL (Table 1). 

**Table 1:** Summarized hematology and blood chemistry WBC, white blood cell count; Hb, hemoglobin; CRP, C-reactive protein; AST, aspartate transaminase; ALT, alanine aminotransferase; CEA, carcinoembryonic antigen

Labs	Normal level	On admission	First week	Second week	Third week
Blood glucose	70-99 mg/dlL	129 mg/dL	111 mg/dL	177 mg/dL	202 mg/dL
WBC	3500-11000/uL	14000/uL	12000/uL	10000/uL	15600/uL
Hb	12-16 g/dL	9.2 g/dL	9.0 g/dL	9.0 g/dL	8.2 g/dL
Platelet	130000-400000/uL	130000/uL	25000/uL	58000/uL	110000/uL
CRP	< 5 mg/L	80 mg/L			
AST	≤34 U/L	64 U/L	55 U/L	43 U/L	44 U/L
ALT	7-52 U/L	42 U/L	50 U/L	71 U/L	44 U/L
Sodium	134-148 mEq/L	139 mEQ/L	140 mEQ/L	146 mEQ/L	144 mEQ/L
Potassium	3.6-5.0 mEq/L	2.6 mEq/L	3.6 mEq/L	4.5 mEq/L	4.3 mEq/L
Troponin-I	5-11.8 pg/mL	15.1 pg/mL			
Albumin	3.5-5.7 g/dL	2 g/dL	1.7 g/dL	1.7 g/dL	1.7 g/dL
Calcium	8.6-10.3 mg/dL	7.8 mg/dL	7.5 mg/dL	8.2 g/dL	8.4 mg/dL
Cortisol in am	6.7-22.6 ug/dL	12.10 ug/dL			
Carcinoembryonic antigen	0.0-4.7 ng/mL	26.6 ng/mL	24.6 ng/mL		28.6 ng/mL

**Figure 1:**
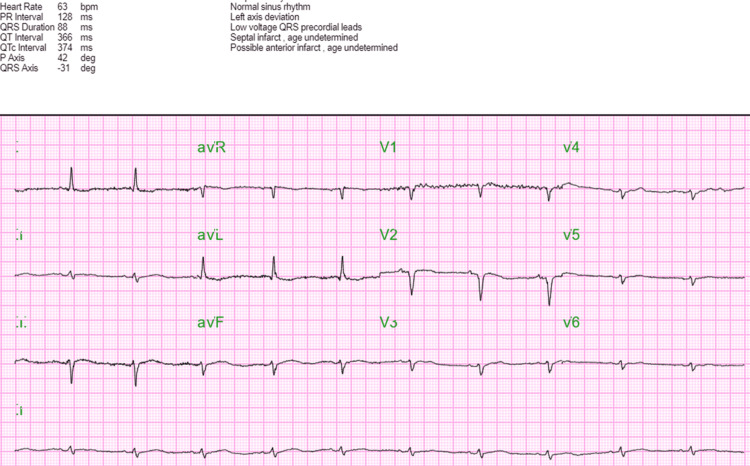
Electrocardiogram taken on the day of admission

Her electrocardiogram demonstrated a normal sinus rhythm with a heart rate of approximately 64 (Figure 1).

**Figure 2:**
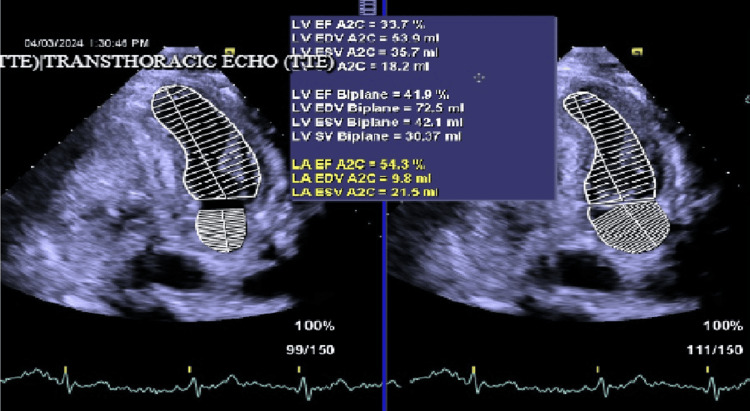
Transthoracic echocardiogram showing EF LV, left ventricular; LA, left atrium; EF, ejection fraction; EDV, end-diastolic volume; ESV, end-systolic volume

The chest X-ray film shows no acute consolidation. Her echocardiogram showed ejection fraction (EF) of 45% (new cardiac heart failure with reduced EF) (Figure 2).

Elevated prothrombin time (PTT) due to factor XI and XII deficiency (Table 2). 

**Table 2:** Coagulation profile on hospital stay PTT, prothrombin time; INR, international normalized ratio

Coagulation profile	Normal range	On hospital stay	Recent
PTT	24.9-34.9 sec	247-170 sec	32.1 sec
INR	0.85-1.15	1.24-1.92	1.53
Factor XI	60-150%	40-51	50
Factor XII	50-150%	40-49	49

Based on the test results mentioned and the occurrence of hypothermia alongside altered mental status and anasarca, suspicion of myxedema coma was heightened. Hypothyroidism was then confirmed, thyroid function test showed thyroid-stimulating hormone (TSH) of 33.777 uIU/mL, and a low free thyroxine (FT4) of 0.71 ng/dL (Table 3). 

**Table 3:** Summarized thyroid profile TSH, thyroid stimulating hormone; FT4, free thyroxine; T3, triiodothyronine

Parameter	Normal range	Hospital stay	Recent
TSH	0.450-5.350 uIU/mL	33.77-2.8 uIU/mL	2.8 uIU/mL
FT4	0.60-1.60 ng/dL	0.43-1.35 ng/dL	1.35 ng/dL
T3	71-180 ng/dL		66 ng/dL

**Figure 3:**
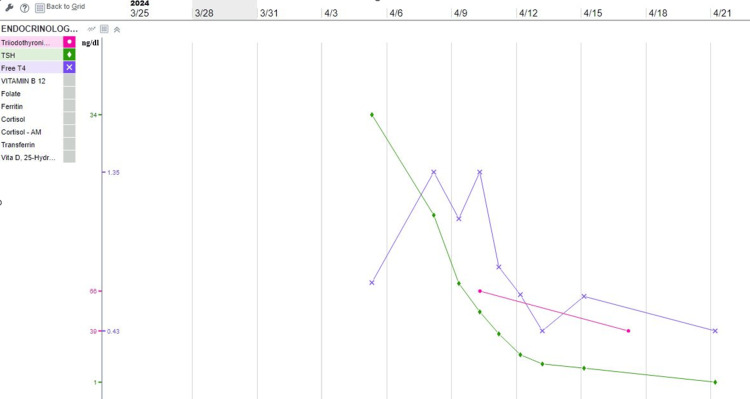
Illustration of thyroid function throughout hospitalization Pink: triiodothyronine; green: TSH; purple: FT4 TSH, thyroid stimulating hormone; FT4, free thyroxine

The patient received levothyroxine 200 mcg IV bolus followed by 100 mcg IV daily. She also received liothyronine 5 mg IV bolus followed by 2.5 mg IV Q8H until clinical improvement. The trend of TSH and FT4 was followed (Figure 3).

Before replacing thyroid hormones, she was started on intravenous hydrocortisone 100 mg q 8 hourly. A blood transfusion was administered because of severe anemia. She received metronidazole and ceftriaxone for leg cellulitis. She was rewarmed using warming blankets, and her hypothermia was resolved within a day.

## Discussion

The term "myxedema coma" is actually a misnomer, as neither coma nor myxedema is necessary for diagnosis. However, as well-defined diagnostic criteria are lacking, we diagnose cases based on the presence of hypothermia, hyponatremia, and/or hypercapnia [7]. 

Treatment for myxedema coma involves thyroid hormone therapy and intensive supportive care. Recent studies recommend combined T4 (levothyroxine) and T3 (triiodothyronine and liothyronine) therapy over T4 alone. Intravenous doses typically start with T4 at 200 to 400 mcg initially, followed by 50 to 100 mcg daily until oral intake is possible. Additionally, an initial intravenous dose of T3 ranges from 5 to 20 mcg, followed by 2.5 to 10 mcg every eight hours until clinical stabilization, with discontinuation thereafter [1,6].

High-dose glucocorticoids, such as intravenous hydrocortisone at 100 mg every eight hours for two days, followed by lower doses, are recommended after ruling out adrenal insufficiency [6].

While it is undeniable that T3 offers greater potency in enhancing thyroid hormone levels, administering it carries risks that warrant careful consideration, such as elevating tissue oxygen consumption, which causes a compensatory rise in cardiac output. This concern is particularly pertinent for elderly patients and those with cardiac comorbidities [8].

Monitoring T4 and TSH levels objectively observe improvement in myxedema coma patients. However, as TSH levels reflect thyroid status over the prior six to eight weeks, they may lack representativeness in assessing thyroid treatment response. Thus, assessing therapy response through clinical improvement alongside T4 status provides superior insights [8]. Myxedema coma may heighten the risk of bleeding due to decreased levels of coagulation factors [9].

In 1965, Simone et al. demonstrated decreased values of factors VIII, IX, and XI in patients with hypothyroidism. Recent findings show increased bleeding time, PTT, and activated partial thromboplastin time (APTT), alongside reduced FVIII activity and von Willebrand factor (VWF) activity in patients with overt hypothyroidism compared to controls. These abnormalities reversed after levothyroxine treatment [10].

In our specific case, the patient's diagnosis revealed deficiencies in both factor XI and factor XII, which present as rather unique findings. The factor levels improved as the treatment for hypothyroidism was administered. This combination of deficiencies is not commonly encountered and may suggest an atypical underlying pathology or a distinctive clinical presentation. Further investigation and monitoring are warranted to fully understand the implications of these coexisting deficiencies and their impact on the patient's overall health and treatment approach.

## Conclusions

We report a case of myxedema coma combined with sepsis and factor deficiency, each posing inherent risks of coagulopathies. Patients in myxedema coma face an increased susceptibility to bleeding complications due to reduced thyroid hormone levels, especially those with coagulation factor deficiencies. Moreover, we can conclude that as FT4 levels increase, so does the concentration of coagulation factors, emphasizing the importance of promptly starting treatment upon suspicion of myxedema coma. Further clinical studies are crucial for a thorough grasp of how thyroid hormone affects hemostasis in patients with factor deficiencies.
